# Financial counselling gaps in Alzheimer's care: Patient out-of-pocket expenses and physician barriers in a regional German cross-sectional pilot study

**DOI:** 10.1016/j.dialog.2026.100364

**Published:** 2026-10-07

**Authors:** Younes Adam Tabi, Eva Christina Meyer, Abdelati Tabi, Julius Rave, Sarah Philippen, Thorsten Bartsch

**Affiliations:** a Department of Neurology, University Medical Centre Kiel, Arnold-Heller-Straße 3, 24105, Kiel, Germany; b Department of Internal Medicine, Schön Klinik Eckernförde, Schleswiger Straße 114, 24340 Eckernförde, Germany; c Associated Researcher

**Keywords:** Alzheimer's disease, Out-of-pocket expenses, Financial counselling, Dementia care, Pilot study

## Abstract

Alzheimer's disease (AD) is associated with substantial societal and household costs, but patient-level direct out-of-pocket expenses and perceived financial counselling needs in routine memory-clinic care remain insufficiently characterised. We conducted a regional exploratory mixed-methods cross-sectional pilot survey in Schleswig-Holstein, Germany, including 38 patients recruited from a single university memory clinic and 136 responding outpatient physicians (general practitioners and neurologists; 8.8% response rate). Patients reported direct out-of-pocket expenses, perceived financial impact, and information/counselling needs. Physicians reported counselling practices, perceived burden, and barriers, complemented by brief free-text responses analysed using inductive qualitative content analysis informed by Grounded Theory coding principles.

In this patient sample, 34% reported increased out-of-pocket expenses, most commonly medication co-payments and transportation costs. Nearly 40% reported insufficient information about financial implications and 32% expressed a strong desire for more financial counselling. Bayesian Kendall analyses indicated that higher household income was associated with better perceived health (τ = 0.39, 95% CrI [0.13, 0.58], BF₁₀ = 28.68) and perceived health with quality of life (τ = 0.80, 95% CrI [0.51, 0.90], BF₁₀ = 5.62 × 10^8^). Longer time since symptom onset was associated with lower perceived health (τ = −0.33, 95% CrI [−0.52, −0.08], BF₁₀ = 8.40) and quality of life (τ = −0.38, 95% CrI [−0.56, −0.13], BF₁₀ = 25.64), whereas evidence for an association with total out-of-pocket expense category was inconclusive (τ = 0.20, 95% CrI [−0.04, 0.40], BF₁₀ = 0.86). Among responding physicians, 64% reported rarely or never discussing financial aspects of AD care. Free-text responses described time constraints, limited training/resources, and uncertainty about professional role boundaries.

Thus, modest direct out-of-pocket expenses in this local sample coexisted with substantial perceived information and counselling needs. Because of the single-clinic patient sample and low physician response rate, these findings are exploratory and should not be interpreted as representative of German AD care.

## 1 Introduction

Alzheimer's disease (AD) is the most common form of dementia and progressively impairs memory, cognition, and independence [1], [2]. With population aging, the number of people living with dementia is projected to rise substantially worldwide [3]. Disease-modifying anti-amyloid therapies have emerged, but they do not cure AD and require careful patient selection and monitoring [4]. Alongside its clinical consequences, AD is therefore relevant to patients, families, healthcare systems, and society from an economic perspective.

The broader economic burden of AD is substantial. Global modelling projects marked growth in dementia-related societal burden as populations age, with particularly large future effects in low- and middle-income countries [5]. A narrower and clinically tractable component of this broader burden is direct out-of-pocket expenditure, defined here as payments made by patients or households for medical or non-medical items that are not reimbursed by insurance or other payers. In oncology, distress related to such costs is often discussed under the term “financial toxicity” [6], [7].

European cost-of-illness studies likewise encompass a much broader set of costs than those assessed here. In the GERAS study, average monthly societal costs per patient were approximately €1881 in France, €2349 in Germany, and €2016 in the UK, with informal care accounting for 50–61% of total costs [8]. Such estimates demonstrate that AD can impose substantial formal- and informal-care costs.

Caregiver burden is also clinically important. Prior studies have associated dementia caregiving with depressive symptoms, lower life satisfaction, patient dependency, greater caregiving time, and caregiver distress [9], [10].

Direct patient expenses may become increasingly relevant as AD care pathways evolve. For example, anti-amyloid treatment can require repeated treatment visits and monitoring, potentially adding transportation and other direct patient-level costs. Because healthcare financing differs between countries and because these broader costs were not measured here, the present study does not estimate their magnitude. Instead, we ask whether patients in one regional memory-clinic sample report direct out-of-pocket expenses or unmet financial-information needs, and whether responding outpatient physicians perceive and address such issues in routine care.

Against this background, the present study was designed as a regional exploratory pilot in Schleswig-Holstein, Germany. It does not aim to estimate the total economic burden of AD or national German AD care costs. Rather, it focuses on direct patient-reported out-of-pocket expenses, perceived financial impact, and perceived financial information/counselling needs among patients recruited from a single university memory clinic, alongside physician-reported counselling practices and perceived barriers among outpatient general practitioners and neurologists sampled in Schleswig-Holstein.

The survey design was conceptually inspired principally by the German radiotherapy study of Fabian et al. [11], with the subsequent multicentre work [12] providing additional context: Fabian and colleagues used a study-specific questionnaire that separated direct patient costs and income loss from subjective financial difficulties, global health/quality of life, and preferences for cost-related information. Our questionnaires were not copies of that instrument but disease terminology and expense categories were rewritten for the AD context, and parallel physician questionnaires were developed to assess perceived burden, counselling practice, professional role boundaries, barriers, and suggested support resources. Caregivers were not surveyed as an independent respondent group but assistance with patient questionnaire completion was permitted where needed.

The primary objectives were to (i) describe patient-reported AD-related direct out-of-pocket expenses and perceived financial impact in this pilot sample, and (ii) describe perceived unmet need for financial information and counselling. Secondary objectives were to characterise counselling practices among responding physicians and to identify perceived barriers and suggested improvements from free-text responses.

## 2 Methods

We performed a regional exploratory mixed-methods cross-sectional pilot survey to characterise patient-reported direct out-of-pocket expenses, perceived financial impact, and information/counselling needs in AD, and to assess outpatient physicians' counselling practices and perceived barriers within routine care in Schleswig-Holstein, Germany. The design combined structured questionnaire data with inductive qualitative content analysis informed by Grounded Theory coding principles.

### 2.1 Participant recruitment

#### 2.1.1 Patients

Participants were recruited from the Memory Clinic of the Department of Neurology at the University Hospital Schleswig-Holstein in Kiel, Germany. A total of 80 patients who had recently received a diagnosis of AD were invited to participate in the study. The inclusion criteria were as follows:

The diagnosis of AD was confirmed using established clinical criteria, including the National Institute on Aging–Alzheimer's Association (NIA-AA) guidelines [13] and International Working Group (IWG) criteria [14], based on clinical history, cognitive assessment and, where appropriate, biomarker information. Caregiver assistance with reading and/or completing the questionnaire was permitted where needed. Whether an individual questionnaire was completed independently or with caregiver assistance was not recorded, so patient-alone versus caregiver-assisted completion counts cannot be reconstructed.

Only patients whom the treating neurologist judged capable of providing written informed consent were approached. Capacity was assessed clinically, without a structured capacity instrument. The treating neurologist considered whether the patient understood the study and its procedures, appreciated that participation was voluntary, understood the intended research use of the data, and could communicate a stable participation decision, together with overall clinical judgement. Patients judged unable to provide informed consent were not approached. Because screening was restricted to patients considered capable, the number not approached specifically because of insufficient capacity was not separately recorded. Additional exclusion criteria were severe dementia and language barriers. No formal a priori sample-size calculation was performed because this was an exploratory, hypothesis-generating pilot study.

#### 2.1.2 Healthcare providers

Healthcare providers were sampled from the list of general practitioners and neurologists registered with the Association of Statutory Health Insurance Physicians in Schleswig-Holstein (Kassenärztliche Vereinigung Schleswig-Holstein). Questionnaires were mailed to 1394 GPs and 163 neurologists. Eligible providers had an active practice in general medicine or neurology. No weighting procedures were applied among respondents. The physician results are therefore descriptive of respondents and are not intended as population estimates for physicians in Schleswig-Holstein or Germany.

Separate questionnaires were used for patients, GPs, and neurologists. The patient questionnaire could be completed with caregiver assistance, but caregivers were not surveyed as a separate stakeholder group. The German questionnaires and English translations are provided in the Supplementary Materials.

### 2.2 Variables and operational definitions

All questionnaire items used in the study are reproduced in the Supplementary Materials (patient, GP, and neurologist versions). Analyses were conducted at the item level.

The patient questionnaire was designed to capture a wide range of variables, including:

• Sociodemographic information: age, gender, educational level, household income, and employment status. To reduce participation burden, household income was recorded in predefined ranges rather than as an exact amount, following the general survey approach used by Fabian et al. [11], [12].
• Health Status: Self-reported health status and quality of life, using a 7-point Likert scale.
• Financial Burdens: Specific questions about out-of-pocket expenses related to AD, including medication costs, transportation, and any modifications made to the home environment.
• Awareness and Information: Questions assessing the patients' awareness of the financial implications of their condition and the extent to which they felt informed by their healthcare providers.

The patient questionnaire was conceptually informed principally by Fabian et al. [11], whose study-specific radiotherapy questionnaire assessed direct costs and income loss alongside global health/quality of life, subjective financial difficulties, perceived information, and preferences for cost-related communication. The later multicentre study used the same broader framework [12]. In Fabian et al. [11], the questionnaire itself was designed for that study and pilot-tested in three potentially eligible oncology patients. The financial-difficulties and global health/quality-of-life items drew on the EORTC QLQ-C30, whereas the two information/communication items were not formally validated. We used this item architecture as inspiration rather than transferring an oncology scale to AD. Disease terminology was rewritten for memory disorders, expense categories were adapted to medication/co-payments, transportation, non-reimbursed products and other care-related expenses, and AD-specific information/counselling questions and separate GP/neurologist questionnaires were developed for the present study. Items structurally analogous to the oncology/EORTC questions were analysed descriptively at item level and were not treated as a validated EORTC or financial-toxicity score in AD.

The healthcare provider questionnaire focused on understanding the role of GPs and neurologists in addressing the financial aspects of Alzheimer's care. The questionnaire included the following sections:

• Professional Background: Years of practice, specialization, and frequency of treating Alzheimer's patients.
• Perceptions of Financial Burdens: Likert-scale questions assessing the providers' perceptions of the financial challenges faced by their patients, including the necessity and effectiveness of current support measures. Further, practitioners were asked to rate their perception on whether they should provide financial guidance or not.
• Role in Financial Counselling: Open-ended questions exploring the extent to which providers felt responsible for advising patients on financial matters, barriers to providing this support, and suggestions for improving financial support within the healthcare system.

The resulting questionnaires were not formally psychometrically validated in an AD population. We therefore treat the responses as exploratory item-level data rather than validated scale scores or latent constructs. This distinction is particularly important for employment- and income-related items in a sample in which 89% of respondents were retired. The full German questionnaires and English translations are provided to make the exact wording transparent and to support future validation work.

### 2.3 Measurement details

Perceived health and quality of life were each assessed with single-item 7-point Likert scales (higher values indicating better perceived health/quality of life). Out-of-pocket expenses were assessed by asking whether AD was associated with increased personal expenses, the type of expense (e.g., co-payments, transportation, non-reimbursed medication/products, other care-related expenses), and categorical amount ranges. A separate item asked whether the patient or relatives had experienced income loss. Because this item was sparse and is outside the primary direct-expense focus, it is reported descriptively and not used for confirmatory inference. Information sufficiency and desire for financial counselling were assessed using ordinal response options. Physician items assessed perceived financial burden, counselling responsibility/frequency, perceived effectiveness of support measures, and free-text views on role, barriers, expenses and suggested improvements.

Educational attainment was classified according to the German school-leaving system: Abitur (≈12–13 years; general university entrance qualification), Realschulabschluss (≈10 years; eligibility for many vocational training programs and, depending on federal state/additional qualifications, access to some higher-education pathways), Hauptschulabschluss (≈9 years; primarily qualifying for basic vocational training), and no formal secondary school qualification.

### 2.4 Data collection

Data were collected through mailed surveys. For patients, the questionnaires were mailed to their homes. Healthcare providers received the questionnaires by post to their respective offices. All questionnaires were accompanied by a prepaid return envelope. Data collection occurred between 11/07/2023 and 31/12/2024.

### 2.5 Bias and data quality considerations

Surveys were completed at home and returned anonymously or in pseudonymised form as applicable. Patient self-report may be affected by recall limitations and cognitive impairment. Caregiver assistance was permitted, but assistance status was not recorded, so any resulting proxy influence cannot be quantified. Physician responses may be affected by non-response and social-desirability biases with the very low response rate constituting a major limitation. Reasons for non-participation were not systematically recorded.

### 2.6 Data analysis

#### 2.6.1 Quantitative analysis

Quantitative analyses were performed in Python 3.13.5 using pandas 2.2.3, NumPy 2.3.5, and SciPy 1.17.0. Descriptive statistics summarised participant characteristics, reported expenses, and counselling perceptions. All inferential association analyses reported in the manuscript and Supplementary Materials were estimated within one Bayesian Kendall framework because the key variables were ordinal and non-normal. The observed association measure was tie-adjusted Kendall's τ-b (scipy.stats.kendalltau), and missing observations were excluded pairwise for each association. Bayesian inference followed the test-statistic formulation described by van Doorn et al. [15]. The standardised Kendall statistic was modelled using its asymptotic Gaussian working likelihood under the alternative and combined with a two-sided stretched-beta prior on the latent correlation ρ, with prior width k = 1 and the transformation ρ = sin(πτ/2). The marginal likelihood under the association model was integrated numerically over τ ∈ (−1,1) and compared with the point-null model τ = 0 to obtain BF₁₀. The corresponding posterior density for τ was normalised numerically and used to obtain equal-tailed 95% Bayesian credible intervals (CrI). BF₁₀ values of 1–3, 3–10, 10–30, 30–100, and > 100 were described as anecdotal, moderate, strong, very strong, and extreme evidence for an association, respectively. Values close to 1 were treated as inconclusive, with reciprocal evidence considered when BF₁₀ < 1. Prior sensitivity was evaluated for every inferential association by repeating the analysis at k = 0.25, 0.50, 1.00, 1.50, and 2.00 and by evaluating dense sensitivity curves across k = 0.25–2.00. Complete association estimates, pairwise sample sizes, 95% CrI, and primary BF₁₀ values are reported in Supplementary Table S1. The full selected-width sensitivity results are reported in Supplementary Table S2, with sensitivity curves in Supplementary Figs. S1 and S2. The income-loss item distinguished loss affecting the patient, loss affecting relatives, and no loss; because these categories are nominal and only three respondents reported any loss, this item was summarised descriptively rather than subjected to a rank-correlation analysis. Item availability/missingness is reported in Supplementary Tables S3 and S4.

#### 2.6.2 Qualitative analysis

All available free-text material relevant to the financial-burden and counselling questions was included in the qualitative review. In the patient questionnaire, free-text fields consisted principally of medication specifications and an optional specification accompanying ‘other expenses’. The patient dataset retained 20 medication-name entries, while four patients selected the ‘other expenses’ category. These sparse and item-specific patient entries were reviewed to contextualise expense categories and were not developed into a separate patient thematic framework. The physician questionnaire contained five free-text fields. The collected data contained 65 entries on other perceived expenses; 12 entries on other perceived consequences; 8 entries on other existing support measures/resources; 57 suggestions for improvement; and 99 responses on the physician's perceived role.

Free-text responses were analysed using inductive qualitative content analysis informed by Grounded Theory coding procedures. Coding was conducted by one researcher (YAT); no second coder was involved and intercoder reliability was therefore not calculated. The analysis proceeded from open coding of meaningful units, through axial coding in which related codes were compared and grouped into higher-order categories, to selective integration of the main categories around overarching interpretive themes. Constant comparison was used throughout where newly coded statements were compared with existing codes and categories, and category definitions were refined iteratively where required. For the physician-role question, this process centred on the tension between a primarily medical role and expectations to provide information, coordination or financial guidance. For suggestions for improvement, the categories were integrated around the perceived need for better coordinated and more accessible support.

Because the material consisted of brief survey comments collected at a single time point, we do not claim a formal Grounded Theory in the classical sense as there was no theoretical sampling, no iterative data collection guided by emerging theory, and no claim of theoretical saturation. No prespecified independent recoding pass after finalisation of the coding framework was undertaken. Code stability was therefore operationalised pragmatically during the iterative coding process as the point at which subsequent responses could be accommodated within the existing higher-order categories without requiring a substantively new category. The resulting framework should be understood as Grounded-Theory-informed qualitative content analysis rather than as a fully developed grounded theory. Categories and representative quotations are provided in the Supplementary Materials, German quotations are accompanied by English translations.

### 2.7 Ethics approval and consent

The study was approved by the ethics commission of the Medical Faculty at Kiel University (Ethik-Kommission der Medizinischen Fakultät der Christian-Albrechts-Universität zu Kiel; files A138/17, A 157/07, extension D 501/13; approval of the relevant amendment 10/07/2023). All procedures conformed to the Declaration of Helsinki. All participants who were approached and enrolled provided written informed consent. For patients with AD, the treating neurologist assessed capacity clinically as described in Section 2.1.1. Patients judged unable to provide informed consent were not approached. Patient data were anonymised prior to analysis and physician responses were handled anonymously.

Reporting follows the STROBE guideline for cross-sectional studies. A completed checklist is provided in Supplementary Table S5. STROBE item 13a is addressed by reporting the numbers of patient and physician questionnaires mailed and returned. For item 13b, reasons for non-participation were not systematically collected and therefore cannot be reported. A separate participant flow diagram (item 13c) was not prepared because this mailed cross-sectional survey had a single invitation/return stage and no subsequent enrolment, intervention or longitudinal follow-up/attrition stage. Item-level missingness is reported in Supplementary Tables S3 and S4.

## 3 Results

### 3.1 Sample and response rates

This regional exploratory pilot comprised a convenience sample of patients from one university memory clinic and a self-selected physician respondent sample. A total of 80 patient questionnaires were mailed and 38 were returned (47.5%). Among outpatient physicians, 1557 questionnaires were mailed (1394 to GPs; 163 to neurologists) and 136 were returned (8.8%). Reasons for non-participation were not systematically collected. The percentages and associations reported below describe respondents only and should not be interpreted as population estimates for German AD care.

### 3.2 Patient characteristics

Patients had a mean age of 73.24 years (SD = 8.02; range 58–88), and 55% were female. Most participants lived in two-person households (84%). Educational attainment was Abitur 42%, Realschulabschluss 29%, Hauptschulabschluss 24%, and no formal secondary school education 3%.

Regarding insurance status, 79% reported statutory health insurance and 21% private insurance; among those with statutory insurance, 13% were exempt from co-payments. Most patients were retired (89%), with 8% employed and 3% civil servants. Monthly net household income varied, with 16% reporting <€1300 and 13% reporting >€5000.

### 3.3 Healthcare provider characteristics and contact with Alzheimer's patients

Among the 136 healthcare providers, 84% were GPs and 16% neurologists; 55% were female. Mean age was 52.76 years (SD = 9.37), and 56% had been practicing for more than 10 years. Frequency of face-to-face contact with Alzheimer's patients is summarised in Fig. 1A: approximately one third reported weekly contact, 30% reported more than one contact per week, and 18% reported daily contact.

**Fig. 1:**
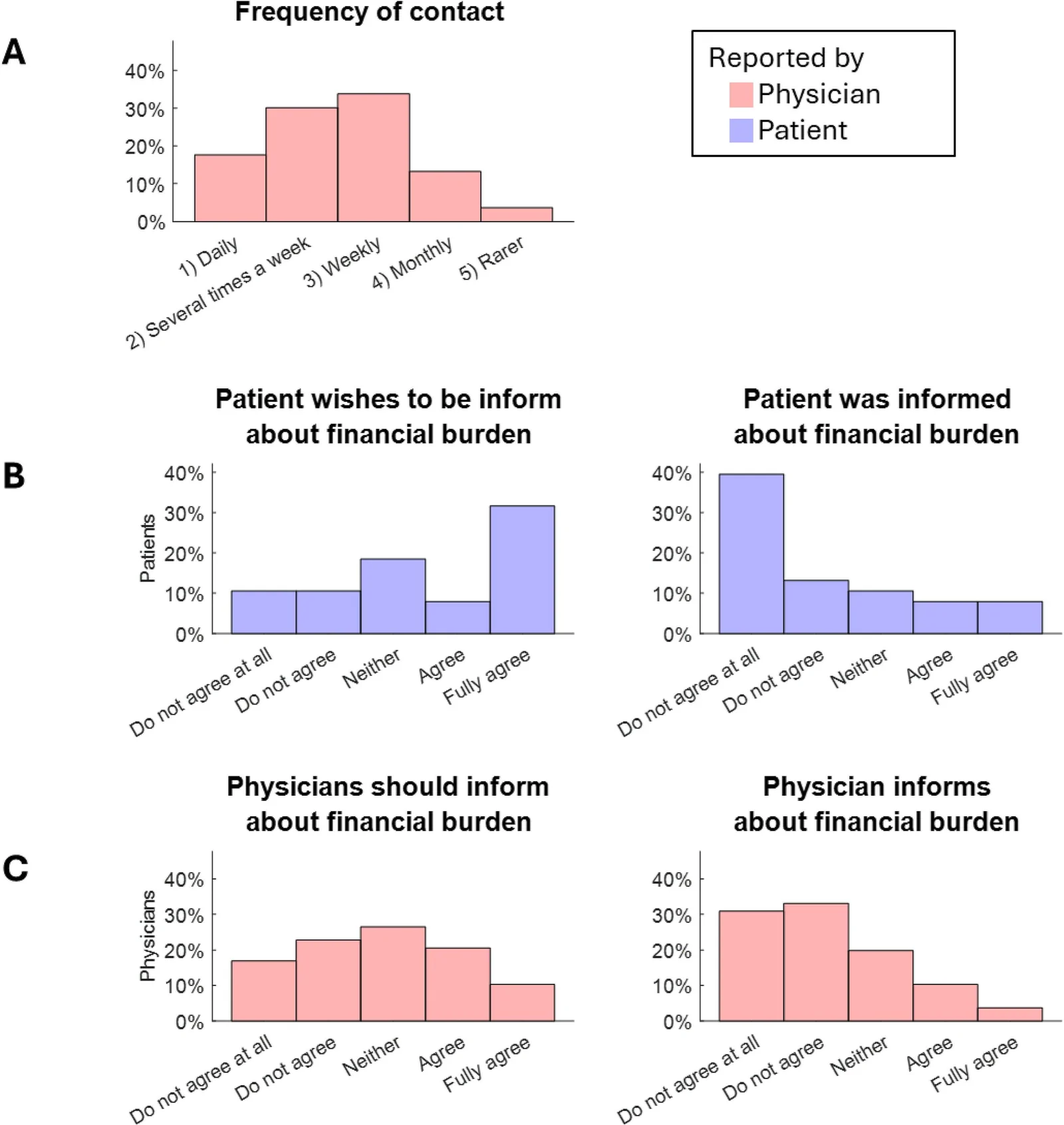
| Survey response distributions. (A) Physician-reported frequency of face-to-face contact with patients with AD (*n* = 136). (B) Patient-reported sufficiency of financial information and desire for financial counselling (*n* = 38). (C) Physician-reported perceived responsibility for and frequency of discussing financial aspects of AD care (*n* = 136). Bars indicate percentages within response categories.

### 3.4 Patient-reported financial burden and unmet need for financial information

Thirteen of 38 patients (34%) reported increased out-of-pocket expenses related to AD, most commonly medication co-payments and transportation to medical appointments. Across the full patient sample, reported total additional expense categories were < €100 for 6/38 (16%), €100–€500 for 3/38 (8%), and €501–€1500 for 4/38 (11%); no respondent reported a higher category. No patient reported a severe effect on household finances; 16% reported a moderate and 13% a mild effect. Three respondents indicated disease-related income loss affecting themselves (*n* = 1) or relatives (*n* = 2); because this item was sparse and caregivers were not independent respondents, it was not used for inferential analyses.

Patients also reported an information gap concerning financial implications. Nearly 40% indicated that they had not received sufficient information, and 32% strongly desired more comprehensive financial counselling (Fig. 1B). These percentages describe this memory-clinic convenience sample rather than a population prevalence of unmet counselling need.

### 3.5 Socioeconomic correlates of perceived health, quality of life, and financial impact

Higher monthly household income was associated with better perceived health (τ = 0.39, 95% CrI [0.13, 0.58], BF₁₀ = 28.68; Fig. 2A), and perceived health was strongly associated with quality of life (τ = 0.80, 95% CrI [0.51, 0.90], BF₁₀ = 5.62 × 10^8^; Fig. 2B). The perceived impact of financial strain on physical health was greater at lower income (τ = −0.41, 95% CrI [−0.59, −0.15], BF₁₀ = 44.93; Fig. 2C) and at lower educational level (τ = −0.33, 95% CrI [−0.52, −0.08], BF₁₀ = 7.70; Fig. 2D).

**Fig. 2:**
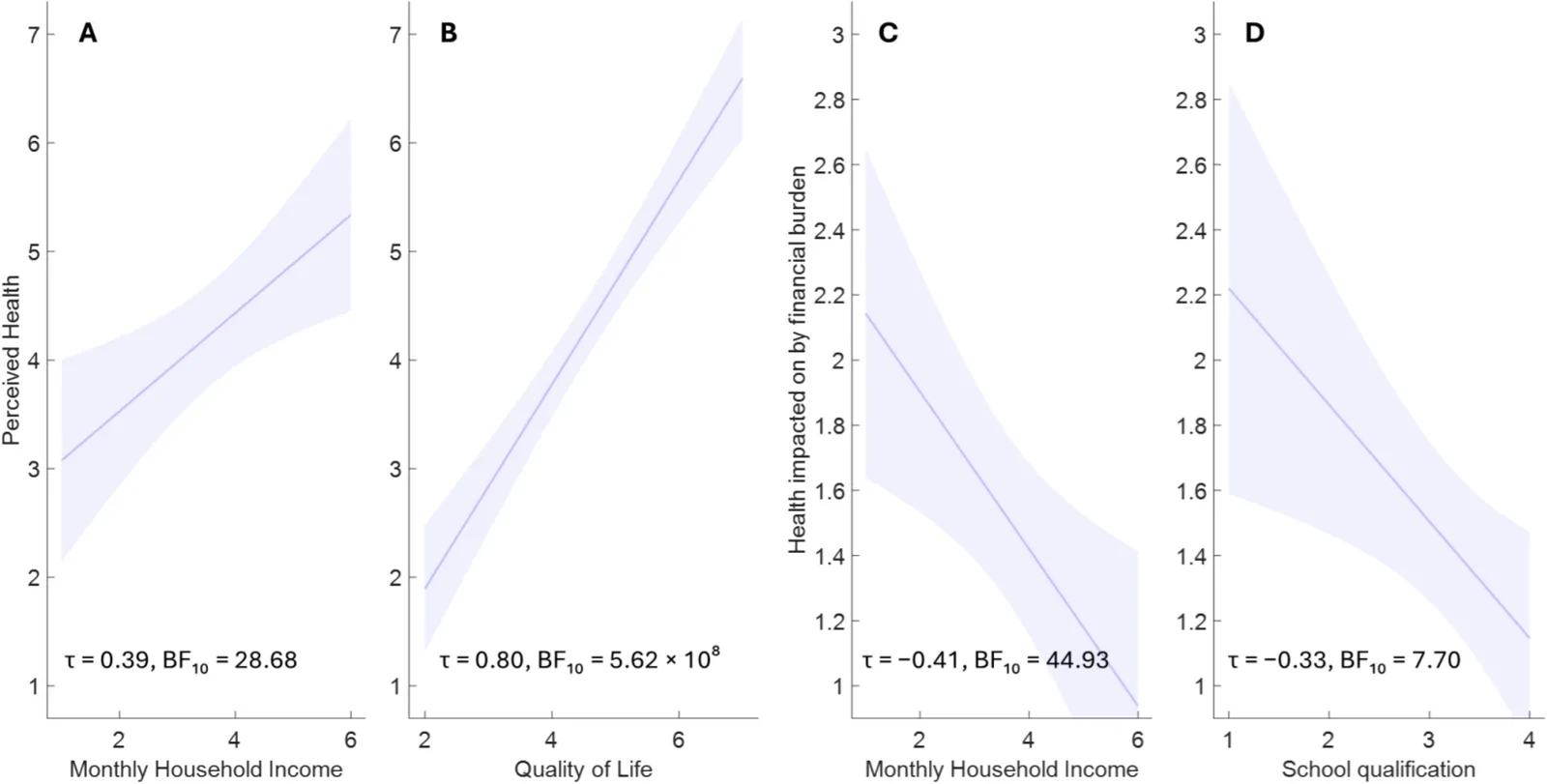
| Patient-level associations between socioeconomic factors, perceived health/quality of life, and perceived financial impact (*n* = 38). Panel annotations show Kendall's τ and BF; pairwise sample sizes and 95% Bayesian credible intervals are reported in the Results and Supplementary Table S1.

### 3.6 Disease duration and exploratory patient-reported outcomes

Patients completed the questionnaire a mean of 37.86 months (SD 27.29; range 6–120) after first symptom onset, not after diagnosis. Longer time since symptom onset was associated with lower perceived health (τ = −0.33, 95% CrI [−0.52, −0.08], BF₁₀ = 8.40) and lower quality of life (τ = −0.38, 95% CrI [−0.56, −0.13], BF₁₀ = 25.64). Evidence for an association with total out-of-pocket expense category was inconclusive (τ = 0.20, 95% CrI [−0.04, 0.40], BF₁₀ = 0.86).

### 3.7 Physicians' counselling practice, perceived burden, and qualitative explanations

Among responding physicians, 64% reported rarely or never addressing financial aspects of AD care with patients (Fig. 1C), although more than 40% considered such counselling important or within their role. Respondents also varied in whether they perceived additional financial burden and in their estimates of its magnitude (Fig. 3A–B); perceived consequences are summarised in Fig. 4. Responding physicians' ratings of the effectiveness of existing support resources are presented in Supplementary Fig. S3.

**Fig. 3:**
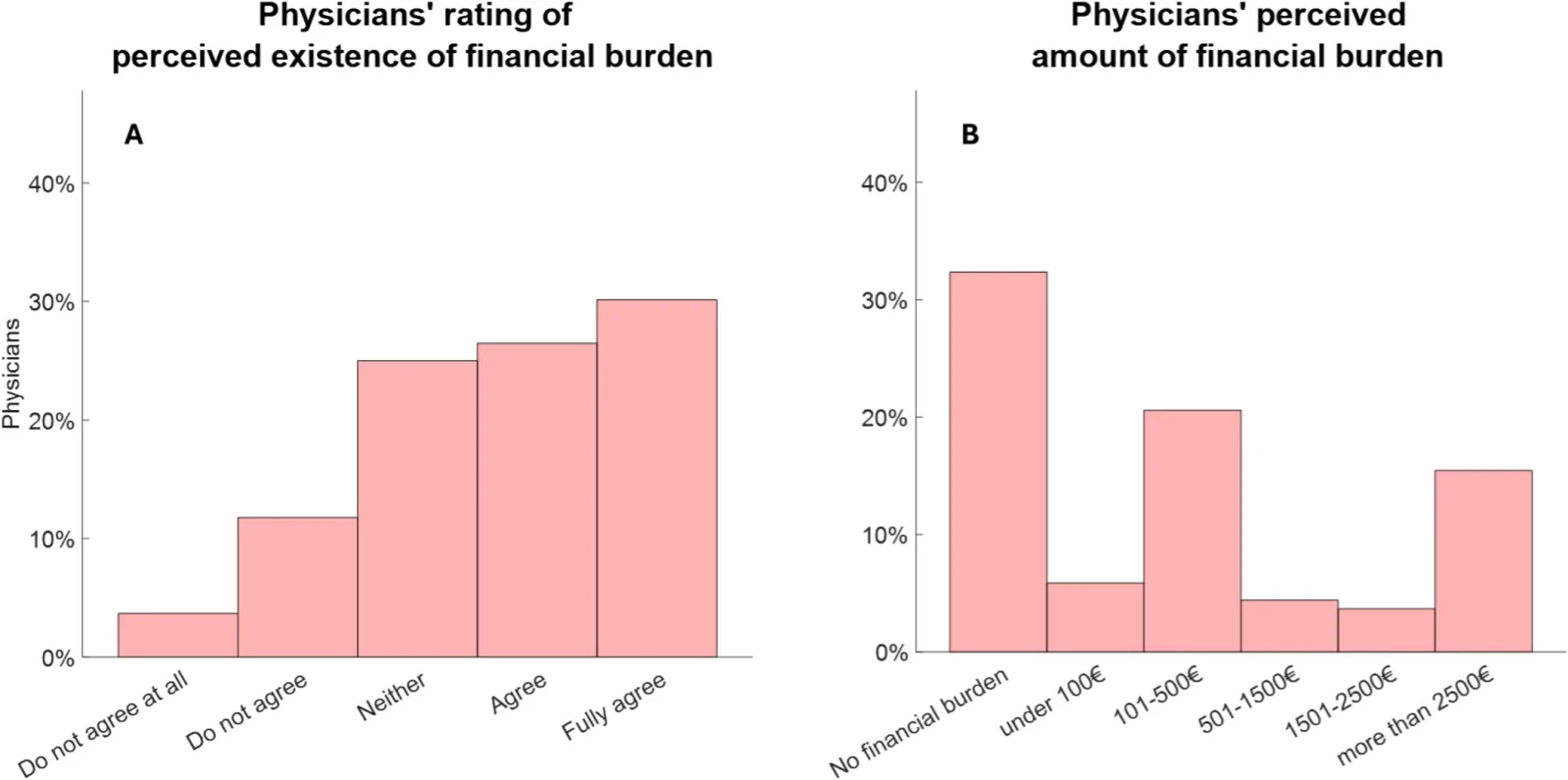
| Physician-reported perceptions of AD-related additional financial burden. (A) Agreement that patients experience additional financial burden. (B) Estimated magnitude of that burden. Bars indicate percentages within response categories (*n* = 136).

**Fig. 4:**
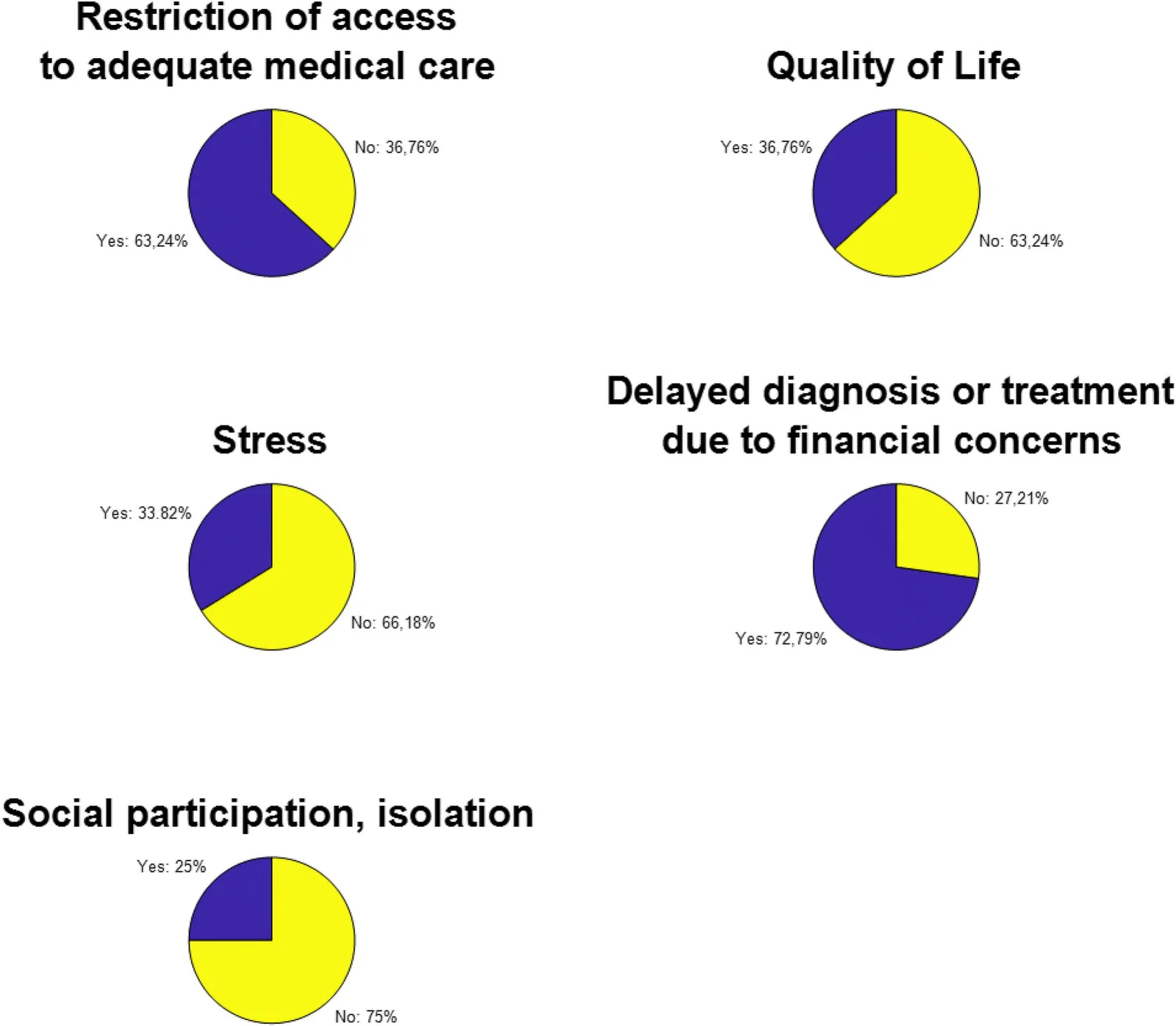
| Physician-reported perceived consequences of additional financial burden. Pie charts show the proportion of respondents endorsing each consequence category (*n* = 136).

The qualitative material was used to contextualise, not statistically validate, this quantitative attitude–practice pattern. Quantitatively, low counselling frequency coexisted with endorsement of counselling relevance among a substantial subgroup. In free text, time constraints mapped onto limited practical capacity to provide counselling, statements about professional role boundaries mapped onto disagreement over whether physicians should provide detailed financial advice, and limited training/resources mapped onto uncertainty about what information to provide and where to refer.

Respondents also suggested potential improvements, including more accessible community and social-care resources, clearer referral pathways, financial relief measures, improved access to specialised services, and stronger support for families. These categories are descriptive summaries of the free-text material and should not be interpreted as prevalence estimates.

### 3.8 Drivers of physicians' counselling efforts and perceived effectiveness of support measures

Physicians' perceived need to provide financial counselling increased with greater perceived likelihood of disease-related additional financial burden (τ = 0.30, 95% CrI [0.18, 0.41], BF₁₀ = 33,171.11; Fig. 5A) and with greater perceived magnitude of that burden (τ = 0.27, 95% CrI [0.14, 0.39], BF₁₀ = 1016.58; Fig. 5B). Longer time in practice was associated with lower perceived need to provide financial counselling (τ = −0.22, 95% CrI [−0.33, −0.10], BF₁₀ = 115.50; Fig. 5C). Physicians who endorsed a greater need for financial counselling were also more likely to report providing it (τ = 0.45, 95% CrI [0.33, 0.55], BF₁₀ = 6.11 × 10^11^; Fig. 5D).

**Fig. 5:**
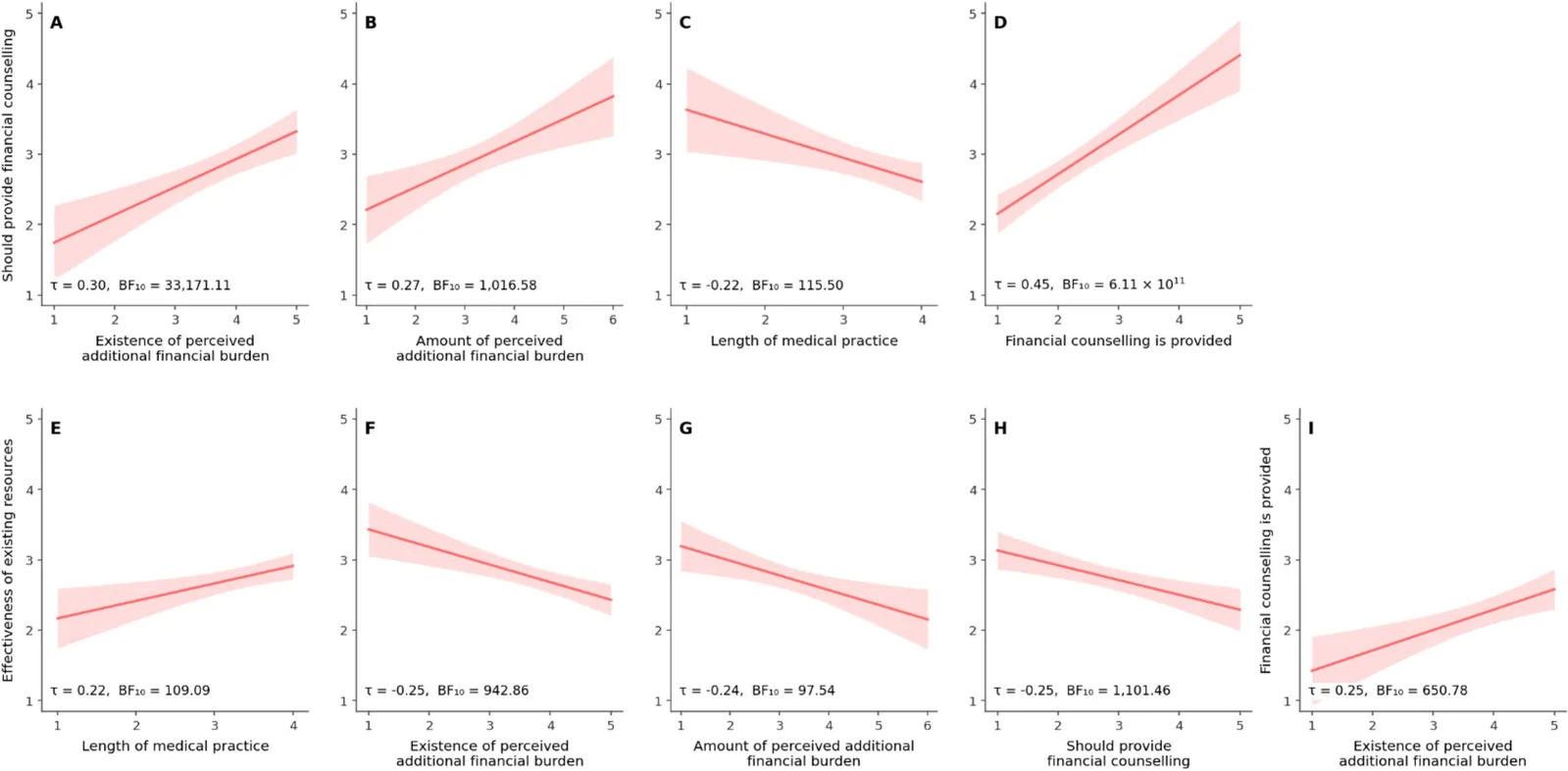
| Associations among physicians' perceptions of additional financial burden, counselling need, counselling provision and perceived effectiveness of support measures (*n* = 136). Panel annotations show Kendall's τ and BF₁₀; pairwise sample sizes and 95% Bayesian credible intervals are reported in the Results and Supplementary Table S1.

Longer time in practice was associated with higher perceived effectiveness of existing support measures (τ = 0.22, 95% CrI [0.10, 0.32], BF₁₀ = 109.09; Fig. 5E). Higher perceived effectiveness was associated with lower ratings of the existence of additional financial burden (τ = −0.25, 95% CrI [−0.36, −0.13], BF₁₀ = 942.86; Fig. 5F), lower perceived burden magnitude (τ = −0.24, 95% CrI [−0.35, −0.11], BF₁₀ = 97.54; Fig. 5G), and lower perceived need to provide financial counselling (τ = −0.25, 95% CrI [−0.36, −0.13], BF₁₀ = 1101.46; Fig. 5H). Finally, stronger endorsement of additional financial burden was associated with more frequent provision of financial counselling (τ = 0.25, 95% CrI [0.13, 0.36], BF₁₀ = 650.78; Fig. 5I). These analyses describe associations among respondents and do not imply causality.

## 4 Discussion

This regional exploratory mixed-methods pilot yielded two complementary observations in the sampled respondents as direct patient-reported out-of-pocket expenses were usually modest in absolute terms, while perceived financial-information and counselling needs were common. At the same time, responding outpatient physicians described substantial practical and role-related barriers to discussing financial issues. These findings should be read as hypothesis-generating rather than representative of German AD care. Within the patient sample, lower income and lower education were associated with a greater perceived health impact of financial strain, suggesting that socioeconomic resources may modify how even relatively limited direct expenses are experienced [16], [17].

### 4.1 Why might out-of-pocket expenses appear relatively modest in this cohort?

One plausible explanation for the comparatively modest direct expenses is the German insurance context, in which statutory health insurance covers many medical costs and co-payment relief mechanisms exist for some patients. In addition, 89% of this sample was retired, making employment-related financial consequences less salient than in working-age cohorts. Even so, recurrent medication co-payments and transportation expenses may matter more to patients with fewer financial resources. This interpretation is consistent with broader evidence linking financial strain and chronic-disease management, but it remains exploratory in the present small sample [18].

Our findings concern direct patient-reported expenses and perceived strain but they should not be interpreted as evidence that the overall economic burden of AD is modest. External cost-of-illness studies show that informal care and other household costs can be substantial [19], [20], but caregiver time costs, productivity loss, and total household costs were not quantified here. They are therefore background context rather than outcomes of this study.

### 4.2 Why does unmet counselling persist despite moderate direct costs?

The quantitative and qualitative strands suggest that unmet counselling may reflect information and navigation needs as much as the absolute amount of direct expenditure. Patients in this sample frequently reported insufficient financial information, while responding physicians often reported that financial topics were not routinely addressed. The physician free text offers plausible contextual explanations – limited consultation time, uncertainty about professional boundaries, and limited knowledge of referral resources.

### 4.3 Reconciling the physician attitude-practice gap

Although many responding physicians endorsed financial counselling as relevant, most reported limited routine engagement with it. The free-text categories suggest a structural mismatch between perceived relevance and feasible clinical workflows. Respondents described time constraints, a primarily medical role, uncertainty about financial expertise, and the need for clearer referral options. Similar barriers have been reported in other chronic-disease settings [21]. In AD care, any attempt to normalise cost-related communication would therefore need to be brief, role-appropriate, and connected to non-physician support structures rather than assuming that physicians should deliver detailed financial advice themselves.

Notably, providers with more years of practice tended to view existing support measures as more effective, while those perceiving higher patient burden more often endorsed the need for improved support. This pattern may reflect experiential familiarity with local resources among senior clinicians, but it also suggests that resource awareness and referral confidence could be actionable intervention targets in the future.

### 4.4 Potential implications for dementia care pathways

Because this was a regional pilot, the following implications should be treated as testable service-design hypotheses rather than recommendations derived from representative data:

1. Brief screening prompts in outpatient visits (e.g., one question on financial strain and one on information needs), to identify who needs support without extending visits substantially.
2. Standardised information packages (print/online) provided at diagnosis and reinforced at follow-up, tailored to typical cost drivers (transportation, co-payments, care levels, benefits).
3. Clear referral pathways to established advisory structures (e.g., social work, care advisory centres, insurance/care funds counselling), so that physicians can efficiently signpost rather than deliver detailed financial guidance.
4. Targeted training for clinicians focused on ‘what to ask, what to provide, where to refer,’ rather than expecting full financial expertise, consistent with barriers described by physicians in our sample and prior work [21].

A multidisciplinary model may be particularly suitable for testing these hypotheses. Social workers and care-advisory services can potentially provide detailed guidance on benefits, subsidies and practical support, while clinicians could identify patients with information needs and signpost them efficiently [22]. Patient organisations may also help disseminate accessible information. Pharmacists were not surveyed here but their potential role in medication-cost education or signposting therefore remains a hypothesis for future system design rather than an inference from our sampled stakeholders [23], [24].

### 4.5 Limitations

Although this study explores an under-researched aspect of AD, it has several limitations. First, the patient sample was small (*n* = 38) and recruited by convenience from a single university memory clinic, limiting statistical precision and external validity. The sample may over-represent patients who are able to access specialist diagnostic care. Second, the physician response rate was only 8.8%, creating a risk of non-response bias. Respondents may differ from non-respondents in workload, interest in dementia, familiarity with support services, or willingness to discuss financial issues. Consequently, no result should be interpreted as a population estimate for German patients or physicians. Third, the cross-sectional design precludes causal inference.

Fourth, the questionnaires were developed for this exploratory study using prior oncology surveys as conceptual inspiration but were not psychometrically validated in AD. Their measurement properties – including reliability and construct validity – are therefore uncertain, and some employment-related items have limited applicability in a cohort that was 89% retired. Fifth, patient self-report may be affected by cognitive impairment and recall error. Caregiver assistance was permitted but not recorded at the individual-questionnaire level, preventing separation of independent from assisted reports. Finally, although open, axial and selective coding procedures were used to organise and integrate the material, the resulting categories should be interpreted as Grounded-Theory-informed descriptive themes rather than as a formally saturated grounded theory.

### 4.6 Looking ahead: Novel antibody therapies and the risk of widening inequities

Disease-modifying therapies such as lecanemab and donanemab may alter the resource profile of AD care because treatment can require repeated visits and monitoring [25], [26], [27]. Such care pathways could plausibly add direct patient expenses such as transportation. The present finding that lower-income respondents reported greater perceived health impact of financial strain supports evaluating equity explicitly when these therapies are implemented more broadly, while avoiding assumptions about the magnitude of unmeasured household or caregiver costs [16], [17].

### 4.7 Conclusion and suggestions for future research agenda

In summary, this regional exploratory pilot found generally modest direct patient-reported out-of-pocket expenses alongside substantial perceived gaps in financial information and counselling, while responding physicians described practical and role-related barriers to addressing these issues. As the results are not representative estimates of German AD care, larger multi-site studies should evaluate whether brief screening plus structured referral pathways improve access to appropriate support. Longitudinal work will also be needed to determine whether patient-level expenses and counselling needs change as higher-resource disease-modifying treatment pathways become more common.

## CRediT authorship contribution statement

**Younes Adam Tabi:** Writing – original draft, Visualization, Validation, Project administration, Methodology, Investigation, Formal analysis, Data curation, Conceptualization. **Eva Christina Meyer:** Writing – review & editing, Project administration, Methodology, Investigation, Data curation, Conceptualization. **Abdelati Tabi:** Writing – review & editing, Project administration, Methodology, Investigation, Data curation, Conceptualization. **Julius Rave:** Writing – review & editing, Project administration, Methodology, Data curation. **Sarah Philippen:** Writing – review & editing, Project administration, Data curation. **Thorsten Bartsch:** Writing – review & editing, Validation, Supervision, Project administration, Methodology, Investigation, Funding acquisition, Conceptualization.

## Declaration of competing interest

The authors declare that they have no known competing financial interests or personal relationships that could have appeared to influence the work reported in this paper.
