# Supplementary material for: Financial counselling gaps in Alzheimer's care: Patient out-of-pocket expenses and physician barriers in a regional German cross-sectional pilot study
Source: Dialogues Health. 2026 Oct 7;9:100364. doi: 10.1016/j.dialog.2026.100364 (PMC13691146; doi:10.1016/j.dialog.2026.100364)
Supplement: Supplementary file 1 [file mmc1.docx]

**Supplementary Materials:**

***Inductive qualitative content analysis of physicians’ perceived role in financial counselling***

Initial coding and representative quotations:

1. *Time Constraints:*
   - "Viel Zeit in Anspruch" [Takes up a lot of time]
   - "Zeitmangel" [Lack of time]
   - "50 Patienten pro Vormittag" [50 patients per morning]
   - "keine Zeit dafür" [No time for that]
   - "zeitraubend" [Time-consuming]
   - "zeitlich und betriebswirtschaftlich nicht umsetzbar" [Not feasible in terms of time and business operations]
   - "zu wenig Zeit" [Too little time]
2. *Non-Medical Role of GPs:*
   - "Nicht Teil der ärztlichen Arbeit" [Not part of the medical work]
   - "Keine ärztliche Aufgabe" [Not a medical task]
   - "finanzielle Themen sind keine Hausarztbereiche" [Financial matters are not the responsibility of a general practitioner]
   - "unsere Aufgabe ist in erster Linie Diagnostik und Therapie" [Our primary task is diagnostics and therapy]
   - "nicht der erste Ansprechpartner für finanzielle Aspekte" [Not the primary contact for financial aspects]
3. *Need for Specialized Support:*
   - "Einbindung Familie, Ansprechpartner" [Involvement of family, points of contact]
   - "Einbindung in ehrenamtliche Netzwerke" [Involvement in volunteer networks]
   - "Sozialarbeiter, Beratungsstellen" [Social workers, counseling centers]
   - "Vernetzung mit Beratungsstellen, Pflegekassen" [Networking with counseling centers, care funds]
   - "Verweisen auf entsprechende vorhandene Beratungs- und Unterstützungsangebote" [Referral to existing counseling and support services]
4. *Provision of Information:*
   - "Über zusätzliche Unterstützungsmöglichkeiten informieren" [Inform about additional support options]
   - "Stellungnahme, Zuführung zu Hilfsangeboten" [Statement, referral to support services]
   - "Weitergabe von Informationen, Hilfe mit Formularen" [Provision of information, help with forms]
   - "Informationen erteilen/aushändigen" [Provide/distribute information]
   - "Beratung über Zuzahlungsbefreiung" [Counseling about exemption from co-payments]
   - "Hinweise, Atteste" [Advice, certificates]
5. *Role of GPs in Support:*
   - "Beratung der Angehörigen" [Counseling of relatives]
   - "Beratung der Patienten und Angehörigen über Hilfsangebote" [Counseling patients and relatives about support options]
   - "Hilfe bei Anträgen" [Help with applications]
   - "Beratung über Möglichkeiten, Gespräche mit Angehörigen/Bevollmächtigten" [Counseling about options, discussions with relatives/authorized persons]
6. *Challenges and Limitations:*
   - "Wenig Ressourcen" [Few resources]
   - "zu viele Sitze nicht besetzt" [Too many positions unfilled]
   - "Finanzielle Angelegenheit ist keine ärztliche Tätigkeit" [Financial matters are not a medical activity]
   - "unrealistisch, so schon sehr zeitaufwendige Behandlung" [unrealistic, already a very time-consuming treatment]
7. *Suggestions for Improvement:*
   - "Mehr Zeit für Patienten und Angehörige" [More time for patients and relatives]
   - "Mehr Anlaufstellen" [More points of contact]
   - "Flyer wäre gut" [A flyer would be good]
   - "Ärzte sollten besser geschult werden" [Doctors should be better trained]

Axial coding / higher-order categories:

- **Time and resource constraints: limited consultation time, high workload, and insufficient resources.**
- **Professional role boundaries: a primarily medical role contrasted with expectations for financial guidance.**
- **Coordination and referral: perceived need to connect patients with specialised services and advisory structures.**
- **Information provision: provision of basic information and guidance to patients and families.**
- **Support and advocacy: practical help with applications, documentation, and communication with relatives/authorised persons.**
- **System-level barriers: structural limitations and perceived lack of training in financial matters.**

Selective integration:

Through selective integration, these categories were organised around a recurring interpretive theme: tension between a primarily medical role and expectations to provide or coordinate financial information. This theme integrates the survey categories but is not presented as a formally saturated grounded theory.

***Inductive qualitative content analysis of physicians’ suggestions for improving support***

*Initial coding and representative quotations:*

1. *Support for Family Members:*
   - "Die Familienangehörigen benötigen definitiv mehr Unterstützung" [Family members definitely need more support]
   - "Stiftungen, steuerliche Erleichterungen für Angehörige" [Foundations and tax relief for relatives]
   - "Entlastung von pflegenden Angehörigen ermöglichen" [Enable relief for caregiving relatives]
   - "Mittel für kurzfristige Entlastungen von Angehörigen" [Resources for short-term respite for relatives]
2. *Institutional and Community Support:*
   - "Eine Anlaufstelle zur Beratung" [A central point of contact for counselling]
   - "Mehr Hilfsmöglichkeiten schaffen außerhalb der Pflegedienste" [Create more support options outside formal nursing services]
   - "Tagespflege sinnvoll" [Day care is useful]
   - "Vergleichbares zu Familienentlastendem Dienst" [Something comparable to a family-relief service]
   - "spezielle Krankenschwester/Berater" [Specialised nurse/counsellor]
   - "lokale 'Pflegestützpunkte' multiprofessionell" [Local multiprofessional care-support centres]
   - "Treffen für Betroffene, niedrigschwellig" [Low-threshold meetings for affected people]
   - "gut ausgestattete Tagespflege" [Well-equipped day care]
   - "Mehr Möglichkeiten für Tagespflege" [More day-care options]
   - "Betreute Gruppen mit Angeboten von körperlichen und geistigen Training" [Supervised groups offering physical and cognitive activities]
3. *Financial Relief Measures:*
   - "grundsätzliche Befreiung von der Zuzahlungspflicht" [General exemption from co-payments]
   - "Senkung der Kosten insbesondere bei Heimunterbringung" [Reduce costs, particularly for residential care]
   - "private finanzielle Vorsorge" [Private financial provision]
   - "persönliches Budget wie bei psychiatr. Oder schwerst behandelten Personen" [A personal budget similar to that available for psychiatric or severely affected patients]
   - "steuerliche Entlastungen von Familien" [Tax relief for families]
   - "schnellere und einfachere Zuzahlungsbefreiung" [Faster and simpler exemption from co-payments]
   - "Heimkosten deckeln!" [Cap residential-care costs!]
4. *Systemic and Structural Changes:*
   - "Öffentlichkeit sollte deutliche Zunahme der Erkrankung mehr diskutieren" [The public should discuss the marked increase in the disease more openly]
   - "Früher gab es eine sog. 'Gemeindekrankenschwester'" [There used to be a so-called community nurse]
   - "Erhöhung Pflegegrad/Leistungen aus der Pflegeversicherung" [Increase care-grade recognition/long-term care insurance benefits]
   - "das Geld der Pflegeversicherung wird in sehr vielen Fällen ausschließlich zur monetären Bereicherung beantragt" [In many cases, long-term care insurance money is applied for solely for financial gain]
   - "Anpassung der Kriterien für Pflegegrad abgestimmt auf die Besonderheiten" [Adapt care-grade criteria to the specific features of the condition]
   - "vorhandene Strukturen sollten genutzt und einfacher zugänglich gestaltet werden" [Existing structures should be used and made easier to access]
5. *Specific Suggestions for Services:*
   - "Je Patient Pate/Patin für Behördengänge" [A designated helper for each patient for dealing with authorities]
   - "Übernahme von Taxi (Transport)-Kosten" [Coverage of taxi/transport costs]
   - "Tageskliniken" [Day clinics]
   - "Fahrdienst/Taxischein" [Transport service/taxi authorisation]
   - "rascher höhere Pflegestufe" [Faster assignment to a higher care grade]
   - "Tagespflege, Selbsthilfegruppen für Angehörige" [Day care and self-help groups for relatives]
   - "Fachärztliche Anbindung, Terminvergabe Neurologe dauert zu lange" [Better specialist access; neurology appointments take too long]

Axial coding / higher-order categories:

The initial codes were consolidated into the following broader descriptive categories:

- Family and caregiver support: calls for greater practical, financial, and respite support for relatives and caregivers.
- Institutional and community resources: proposals for accessible day care, consultation points, specialised care, and community-based support.
- Financial relief and policy adjustments: proposals for subsidies, caps on care costs, co-payment relief, and simpler access to support.
- System-level change and awareness: suggestions concerning structural reform, public awareness, and long-term care insurance criteria.
- Service enhancement: specific proposals such as transport support, care coordinators, and improved access to specialist services.

Selective integration:

Through selective integration, the suggestion categories were organised around a recurring interpretive theme: the perceived need for more accessible and better coordinated support systems for patients and families. This theme integrates the survey categories but is not presented as a formally saturated grounded theory.

**Additional physician free-text fields**

Other perceived expenses comprised 65 substantive physician responses and were dominated by additional home, ambulatory, day-care or residential-care needs, household help/supervision, care-related aids/materials, and transport. These write-ins were used to contextualise expense categories rather than to estimate patient-incurred costs.

**Supplementary Table S1: Bayesian Kendall association analyses**

| **Population** | **Association** | **n** | **Kendall’s τ-b** | **95% CrI** | **BF₁₀ (k=1)** | **Evidence** |
| --- | --- | --- | --- | --- | --- | --- |
| Patients | Household income – perceived health | 33 | 0.39 | [0.13, 0.58] | 28.68 | Strong |
| Patients | Perceived health – quality of life | 35 | 0.80 | [0.51, 0.90] | 5.62e+08 | Extreme |
| Patients | Household income – perceived health impact of financial strain | 33 | -0.41 | [-0.59, -0.15] | 44.93 | Very strong |
| Patients | Educational attainment – perceived health impact of financial strain | 34 | -0.33 | [-0.52, -0.08] | 7.70 | Moderate |
| Patients | Symptom duration – perceived health | 34 | -0.33 | [-0.52, -0.08] | 8.40 | Moderate |
| Patients | Symptom duration – quality of life | 34 | -0.38 | [-0.56, -0.13] | 25.64 | Strong |
| Patients | Symptom duration – total out-of-pocket expense category | 34 | 0.20 | [-0.04, 0.40] | 0.86 | Anecdotal/inconclusive evidence favouring null |
| Physicians | Perceived existence of additional burden – perceived need to counsel | 128 | 0.30 | [0.18, 0.41] | 33,171.11 | Extreme |
| Physicians | Perceived magnitude of burden – perceived need to counsel | 111 | 0.27 | [0.14, 0.39] | 1,016.58 | Extreme |
| Physicians | Years in practice – perceived need to counsel | 132 | -0.22 | [-0.33, -0.10] | 115.50 | Extreme |
| Physicians | Perceived need to counsel – reported counselling provision | 132 | 0.45 | [0.33, 0.55] | 6.11e+11 | Extreme |
| Physicians | Years in practice – perceived effectiveness of existing support | 134 | 0.22 | [0.10, 0.32] | 109.09 | Extreme |
| Physicians | Perceived effectiveness – perceived existence of additional burden | 130 | -0.25 | [-0.36, -0.13] | 942.86 | Extreme |
| Physicians | Perceived effectiveness – perceived magnitude of burden | 111 | -0.24 | [-0.35, -0.11] | 97.54 | Very strong |
| Physicians | Perceived effectiveness – perceived need to counsel | 131 | -0.25 | [-0.36, -0.13] | 1,101.46 | Extreme |
| Physicians | Perceived existence of additional burden – reported counselling provision | 129 | 0.25 | [0.13, 0.36] | 650.78 | Extreme |

Note. CrI = equal-tailed 95% Bayesian credible interval for Kendall’s τ-b; BF₁₀ = Bayes Factor for the association model relative to the point-null model τ = 0. Pairwise n reflects complete observations for the two variables in each association. Prior-sensitivity results are reported separately in Supplementary Table S2 and Supplementary Figures S1–S2.

**Supplementary Table S2: Bayes Factor prior-sensitivity analyses for all Bayesian Kendall associations**

| **Population** | **Association** | **n** | **k=0.25** | **k=0.50** | **k=1.00** | **k=1.50** | **k=2.00** |  |
| --- | --- | --- | --- | --- | --- | --- | --- | --- |
| Patients | Household income – perceived health | 33 | 25.16 | 30.44 | 28.68 | 25.19 | 22.16 |  |
| Patients | Perceived health – quality of life | 35 | 2.46e+07 | 1.76e+08 | 5.62e+08 | 8.20e+08 | 9.64e+08 |  |
| Patients | Household income – perceived health impact of financial strain | 33 | 36.44 | 46.26 | 44.93 | 39.92 | 35.34 |  |
| Patients | Educational attainment – perceived health impact of financial strain | 34 | 8.61 | 8.98 | 7.70 | 6.53 | 5.64 |  |
| Patients | Symptom duration – perceived health | 34 | 9.26 | 9.74 | 8.40 | 7.13 | 6.17 |  |
| Patients | Symptom duration – quality of life | 34 | 23.39 | 27.65 | 25.64 | 22.37 | 19.62 |  |
| Patients | Symptom duration – total out-of-pocket expense category | 34 | 1.38 | 1.15 | 0.86 | 0.69 | 0.58 |  |
| Physicians | Perceived existence of additional burden – perceived need to counsel | 128 | 37,552.90 | 39,625.77 | 33,171.11 | 27,718.61 | 23,740.88 |  |
| Physicians | Perceived magnitude of burden – perceived need to counsel | 111 | 1,288.83 | 1,261.84 | 1,016.58 | 838.59 | 713.61 |  |
| Physicians | Years in practice – perceived need to counsel | 132 | 176.58 | 153.13 | 115.50 | 93.13 | 78.34 |  |
| Physicians | Perceived need to counsel – reported counselling provision | 132 | 2.91e+11 | 5.40e+11 | 6.11e+11 | 5.66e+11 | 5.11e+11 |  |
| Physicians | Years in practice – perceived effectiveness of existing support | 134 | 168.05 | 145.02 | 109.09 | 87.88 | 73.89 |  |
| Physicians | Perceived effectiveness – perceived existence of additional burden | 130 | 1,291.21 | 1,203.29 | 942.86 | 770.22 | 652.17 |  |
| Physicians | Perceived effectiveness – perceived magnitude of burden | 111 | 141.82 | 127.00 | 97.54 | 79.16 | 66.80 |  |
| Physicians | Perceived effectiveness – perceived need to counsel | 131 | 1,501.64 | 1,403.56 | 1,101.46 | 900.23 | 762.45 |  |
| Physicians | Perceived existence of additional burden – reported counselling provision | 129 | 905.09 | 834.95 | 650.78 | 530.65 | 448.92 |  |

Note. Entries are BF₁₀ values obtained by repeating each Bayesian Kendall analysis with stretched-beta prior widths k = 0.25, 0.50, 1.00, 1.50, and 2.00. All associations with BF₁₀ ≥ 3 under the primary k = 1 specification remained ≥3 at all selected widths. The symptom-duration–out-of-pocket-expense association remained weak/inconclusive across the examined prior range (dense-grid BF₁₀ range 0.58–1.38).

| **Supplementary Table S3: Availability/missingness of key patient analysis variables** | |
| --- | --- |
| **Item** | **Number unavailable/missing** |
| Current occupational status | 0 |
| Monthly household income category | 2 |
| Sick leave | 0 |
| Income-loss item | 0 |
| Total out-of-pocket expense category | 2 |
| Perceived health impact of financial strain | 3 |
| Sufficiency of financial information | 8 |
| Desire for financial counselling | 8 |
| Perceived health | 3 |
| Quality of life | 3 |
| Disease duration | 2 |
| Association-specific sample sizes reflect pairwise complete observations and are reported in Supplementary Table S1. | |

| **Supplementary Table S4: Availability/missingness of key physician analysis variables** | |
| --- | --- |
| **Item** | **Number unavailable/missing** |
| Perceived existence of additional financial burden | 4 |
| Perceived magnitude of financial burden | 24 |
| Perceived effectiveness of existing support | 2 |
| Perceived need for physician financial counselling | 4 |
| Reported provision of financial counselling | 3 |
| Association-specific sample sizes reflect pairwise complete observations and are reported in Supplementary Table S1. | |

**Supplementary Table S5: STROBE checklist table**

| **Item** | **STROBE criterion** | **Where in manuscript** |
| --- | --- | --- |
| 1a | Indicate study design in the title | Title: “regional German cross-sectional pilot study” |
| 1b | Informative and balanced abstract | Abstract |
| 2 | Background/rationale | Introduction (economic context explicitly separated from measured direct out-of-pocket expenses and counselling needs) |
| 3 | Objectives / hypotheses | End of Introduction: “Primary objectives were…” |
| 4 | Present key elements of study design early | Methods opening sentence |
| 5 | Setting, locations, relevant dates | Methods → Data Collection (Kiel; Schleswig-Holstein; 11/07/2023–31/12/2024) |
| 6a | Eligibility criteria, sources and selection | Methods → Participant Recruitment (Patients; Healthcare Providers) |
| 6b | Matching criteria (if matched) | Not applicable |
| 7 | Clearly define outcomes, exposures, predictors, confounders, effect modifiers | Methods → “Variables and operational definitions”; questionnaire bullet lists; questionnaires in Supplementary materials |
| 8 | Data sources/measurement; comparability | Methods → Measurement details + full questionnaires; exploratory item-level instrument and lack of AD psychometric validation stated |
| 9 | Address potential sources of bias | Methods → Bias and data quality considerations; Discussion limitations |
| 10 | Explain how study size was arrived at | Methods → Participant Recruitment: exploratory pilot; no a priori sample-size calculation |
| 11 | Explain handling of quantitative variables | Methods → Quantitative Analysis (ordinal variables → Kendall’s τ; credible intervals for key patient estimates) |
| 12a | Statistical methods, incl. confounding control | Methods → Quantitative Analysis (single Python Bayesian Kendall τ-b framework; prior, posterior interval, BF₁₀, and pairwise missing-data specification) |
| 12b | Methods for subgroups/interactions | Not applicable as there are no subgroups by design |
| 12c | How missing data were addressed | Methods → Quantitative Analysis: missing values excluded pairwise; item-level missingness in Supplementary Tables S3 and S4 |
| 12d | If sampling strategy requires it, describe analytical methods accounting for it | Methods → Healthcare Providers: “No weighting procedures…” |
| 12e | Sensitivity analyses | Methods → Quantitative Analysis; Supplementary Tables S1–S2 and Supplementary Figures S1–S2 (prior-width sensitivity for every inferential Bayesian Kendall association) |
| 13a | Numbers at each stage | Results 3.1: 80 patient questionnaires mailed → 38 returned; 1,557 physician questionnaires mailed → 136 returned |
| 13b | Reasons for non-participation | Reasons for non-participation were not systematically collected; explicitly stated in Methods and Results. |
| 13c | Flow diagram | No separate flow diagram because the mailed cross-sectional survey had a single invitation/return stage and no subsequent follow-up/attrition stage. |
| 14a | Descriptive data/participant characteristics | Results → Patient Demographics; Healthcare Provider Demographics |
| 14b | Indicate missing data for each variable | Supplementary Tables S3 and S4 report item availability/missingness; Supplementary Table S1 reports pairwise n for every association; free-text response counts are reported in Methods |
| 15 | Outcome data: numbers/summary measures | Results sections |
| 16a | Main results: estimates and precision; adjusted/unadjusted and confounders | Results and Supplementary Table S1 report Kendall’s τ-b, 95% Bayesian CrI, BF₁₀, and pairwise n for every inferential association; no adjusted causal estimates are claimed |
| 16b | Report category boundaries when categorising continuous variables | Results and items in questionnaires provided in appendix |
| 16c | Translate relative risks into absolute risk (when relevant) | Not applicable |
| 17 | Other analyses | Not applicable, analyses are described |
| 18 | Summarise key results with respect to objectives | Discussion opening paragraph + Conclusion |
| 19 | Limitations | Discussion → Limitations (selection, non-response, measurement, capacity, caregiver assistance, single-coder qualitative analysis) |
| 20 | Interpretation, considering other evidence | Discussion integrates German system + oncology parallels + antibody therapy outlook |
| 21 | Generalisability | Discussion → Limitations: single-clinic convenience sample and 8.8% physician response rate constrain generalisability |
| 22 | Funding and role of funders | Provided in the title page |

**Supplementary Figure S1 |** Prior-sensitivity analyses for all patient-level Bayesian Kendall associations. Curves show BF₁₀ across stretched-beta prior widths k = 0.25–2.0; points indicate the primary specification k = 1. Horizontal reference lines indicate BF₁₀ = 1, 3, 10, 30, and 100. Selected prior-width values are tabulated in Supplementary Table S2. All patient associations with BF₁₀ ≥ 3 at k = 1 remained ≥3 across the examined prior range; the symptom-duration–out-of-pocket-expense association remained weak/inconclusive (BF₁₀ range 0.58–1.38).

**Supplementary Figure S2 |** Prior-sensitivity analyses for all physician-level Bayesian Kendall associations. Curves show BF₁₀ across stretched-beta prior widths k = 0.25–2.0; points indicate the primary specification k = 1. Horizontal reference lines indicate BF₁₀ = 1, 3, 10, 30, and 100. Selected prior-width values are tabulated in Supplementary Table S2. All displayed physician associations retained BF₁₀ ≥ 3 across the examined prior range.

**Supplementary Figure S3 |** Responding physicians’ ratings of the effectiveness of existing support resources.

**Questionnaire provided to patients – English Translation**

**Questionnaire provided to patients - German**

**Questionnaire provided to Neurologists – English Translation**

**Questionnaire provided to Neurologists - German**

**Questionnaire provided to GPs – English Translation**

**Questionnaire provided to GPs - German**
